# Supplementary material for: pp32 and APRIL are host cell-derived regulators of influenza virus RNA synthesis from cRNA
Source: eLife. 2015 Oct 29;4:e08939. doi: 10.7554/eLife.08939 (PMC4718810; doi:10.7554/eLife.08939)
Supplement: Supplementary file 1. — DOI: http://dx.doi.org/10.7554/eLife.08939.012 [file elife-08939-supp1.docx]

**Supplementary file 1**. Molecular masses of trypsin-digested peptides from IREF-2 proteins and amino acid sequences of IREF-2 proteins corresponding to the tryptic cleavage molecular mass database.

| Predicted amino acid sequences  from database | Amino acid region | Observed  Mass (Da) | Theoretical  Mass (Da) |
| --- | --- | --- | --- |
| **LELSDNR** | **pp32 (69-75)** | **846.4466** | **846.4321** |
| **RIHLELR** | **pp32 (6-12)** | **936.5708** | **936.5743** |
| **ELVLDNSR** | **pp32 (21-28)** | **945.4983** | **945.5005** |
| **KLELSDNR** | **pp32 (68-75)** | **974.5344** | **974.5271** |
| **VSGGLEVLAEK** | **pp32 (76-86)** | **1101.6135** | **1101.6156** |
| **CPNLTHLNLSGNK** | **pp32 (87-99)** | **1467.7413** | **1467.7378** |
| **LLPQLTYLDGYDR** | **pp32 (138-150)** | **1566.8205** | **1566.8168** |
| **LLPQLTYLDGYDRDDK** | **pp32 (138-153)** | **1924.9676** | **1924.9656** |
| **SLDLFNCEVTNLNDYR** | **pp32 (69-75)** | **1972.9134** | **1972.9075** |
| **SLDLFNCEVTNLNDYRENVFK** | **pp32 (69-75)** | **2590.2335** | **2590.2248** |
| **LELSENR** | **APRIL (69-75)** | **860.4481** | **860.4478** |
| **RIHLELR** | **APRIL (6-12)** | **936.5813** | **936.5743** |
| **KLELSENR** | **APRIL (68-75)** | **988.5415** | **988.5427** |
| **DISTLEPLK** | **APRIL (102-110)** | **1015.5465** | **1015.5676** |
| **IFGGLDMLAEK** | **APRIL (76-86)** | **1193.6271** | **1193.6240** |
| **LPNLTHLNLSGNK** | **APRIL (87-99)** | **1420.7903** | **1420.7912** |
| **LLPQLTYLDGYDR** | **APRIL (138-150)** | **1566.8289** | **1566.8168** |
| **SLDLFNCEVTNLNDYR** | **APRIL (117-132)** | **1972.9212** | **1972.9075** |
